# Supplementary material for: Antimicrobial Mechanism of pBD2 against Staphylococcus aureus
Source: Molecules. 2020 Jul 31;25(15):3513. doi: 10.3390/molecules25153513 (PMC7435708; doi:10.3390/molecules25153513)
Supplement: Supplementary file 1 [file molecules-25-03513-s001.pdf]

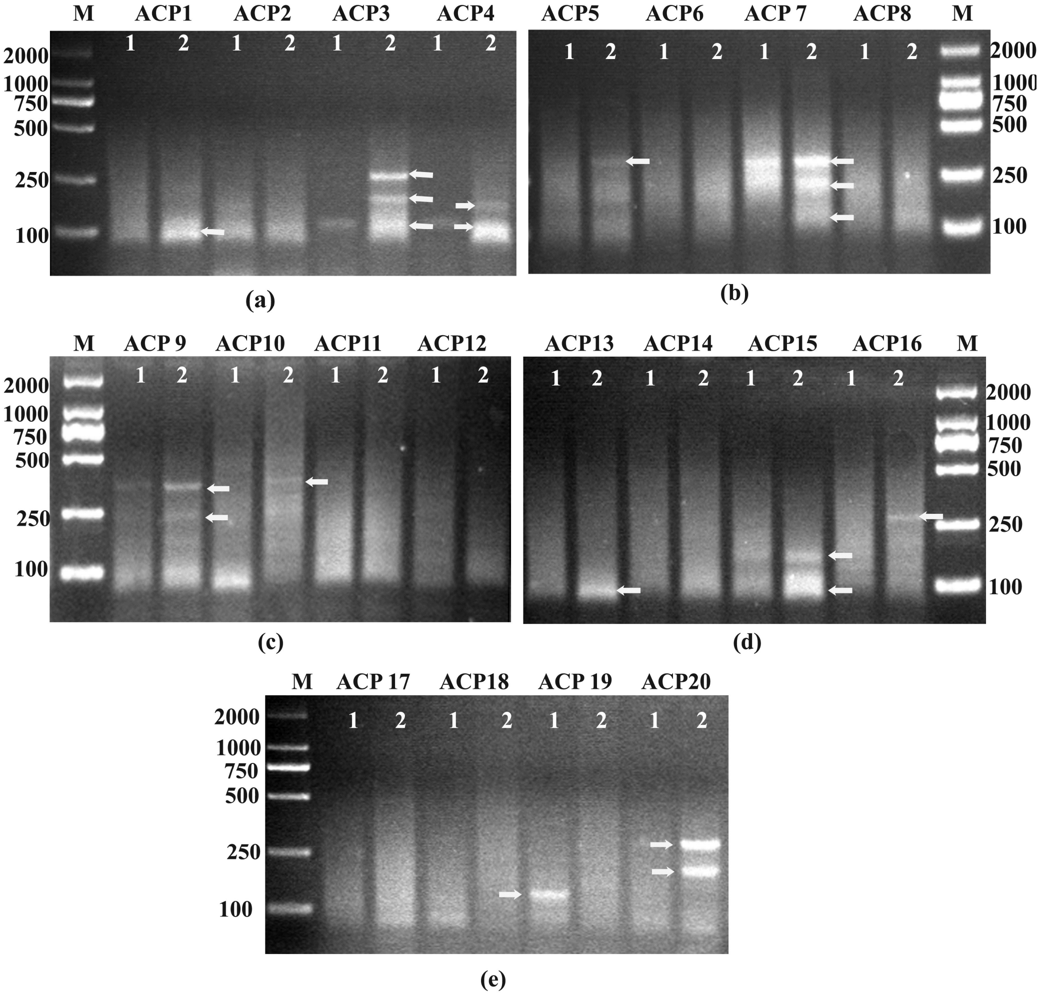

Supplementary Figure 1. Electrophoresis analysis of the DEGs by ACP-based RT-PCR. Agarose gel electrophoresis showed the DEGs between control (lane 1) and bacteria after pBD2-treatment (lane 2) with 20 different arbitrary ACPs. (a),(b),(c),(d) and(e) showed that PCR product with arbitrary ACP1-4, ACP5-8, ACP9-12, ACP13-16, ACP 17-20 respectively; M indicated DNA marker DL2000; The arrows indicated the DEGs.

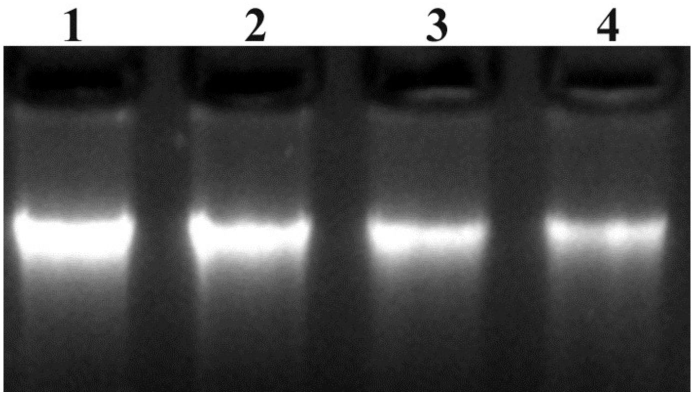

Supplementary Figure2. Gel retardation assay of pBD2 binding to DN**A**. Lane 1-4 indicated that same volume of DNA were incubated with different concentrations of pBD2 (0, 37.5, 75,and 150 μg/mL) for 1.5 h at room temperature, then was electrophoresed on agarose gel for 30 min at 100 V voltage respectively.
